# Acute Exposure to Indoxyl Sulfate Impairs Endothelium-Dependent Vasorelaxation in Rat Aorta

**DOI:** 10.3390/ijms20020338

**Published:** 2019-01-15

**Authors:** Takayuki Matsumoto, Keisuke Takayanagi, Mihoka Kojima, Kumiko Taguchi, Tsuneo Kobayashi

**Affiliations:** Department of Physiology and Morphology, Institute of Medicinal Chemistry, Hoshi University, Shinagawa-ku, Tokyo 142-8501, Japan; k955tkyng@gmail.com (K.T.); cozymihomiho.0210@gmail.com (M.K.); k-taguchi@hoshi.ac.jp (K.T.)

**Keywords:** **Keywords: **aorta, endothelial function, indoxyl sulfate, superoxide dismutase

## Abstract

Gut microbiota are emerging as potential contributors to the regulation of host homeostasis. Dysbiosis of the gut microbiota associated with increased intestinal permeability facilitates the passage of endotoxins and other microbial products, including indoxyl sulfate in the circulation. Although an emerging body of evidence has suggested that indoxyl sulfate is a key substance for the development of chronic kidney disease, few studies have investigated the direct association of indoxyl sulfate with vascular function. We hypothesized that indoxyl sulfate adversely affects vascular function. Aortas isolated from male Wistar rat were examined in the presence or absence of indoxyl sulfate to assess the vascular function, including vasorelaxation and vasocontraction. Indoxyl sulfate (vs. vehicle) (1) decreased vasorelaxation induced by acetylcholine (ACh) but not by sodium nitroprusside; (2) had no significant alterations of noradrenaline-induced vasocontraction in the absence and presence of endothelium; (3) decreased adenylyl cyclase activator (forskolin)-induced vasorelaxation, while such a difference was eliminated by endothelial denudation; and (4) decreased vasorelaxations induced by calcium ionophore (A23187) and transient receptor potential vanilloid 4 agonist (GSK1016790A). The indoxyl sulfate-induced decrease in the vasorelaxations induced by ACh and A23187 increased by cell-permeant superoxide dismutase or by organic anion transporter inhibitor. However, apocynin, an inhibitor of nicotinamide adenine dinucleotide phosphate (NADPH) oxidase, had no effects on vasorelaxations induced by ACh, A23187, forskolin, and GSK1016790A in the presence of indoxyl sulfate. These results suggest that indoxyl sulfate directly affects the vascular function, particularly, endothelium-dependent vasorelaxation, and this effect may be attributable to increased oxidative stress after cell transportion via organic anion transporter, and such increased oxidative stress may not be attributable to activation of NADPH oxidase activation.

## 1. Introduction

Gut microbiota has been implicated in several diseases, including cardiovascular diseases and metabolic diseases [1,2,3,4,5,6,7]. Many substances derived from the gut microbiome, bacterial structural components, and microbial metabolites influence human health and dysregulated homeostasis [5,7,8]. Among them, indoxyl sulfate is derived from the gut microbiotic metabolism of dietary amino acids. Indoxyl sulfate is a protein-bound uremic toxin that is a product of dietary tryptophan metabolism [9,10]. Tryptophan is metabolized into indole by intestinal bacteria (i.e., microbial tryptophanase), and after intestinal absorption, it is sulfated in the liver [5,9]. Indoxyl sulfate has a poor clearance from the systemic circulation in case of impaired renal function [7]; therefore, indoxyl sulfate is present in the circulation in chronic kidney disease (CKD) patients [5,9,11]. In the vascular system, indoxyl sulfate affects various phenomenon, such as the development of calcification [12], inflammation [13,14], vascular smooth muscle cell proliferation [15,16,17], cell senescence [18], and endothelial injury [19].

In addition, endothelial dysfunction has been observed in a uremic circumstance, suggesting the possible role of uremia-associated factors, such as uremic toxins [20]. Indeed, uremic toxins, for example, asymmetric dimethylarginine (ADMA) [21,22], homocysteine [23,24,25], and advanced glycation end products (AGEs) [26,27] and protein-bound uremic toxins, such as indoxyl sulfate and p-cresyl sulfate, have deteriorated vascular tone regulation. Gross et al. [28] found that acute treatment with p-cresyl sulfate augmented phenylephrine (PE)-induced contraction, whereas it had no effect on acetylcholine (ACh)-induced relaxation. Six et al. [29] found that not only acute, but also prolonged treatment with indoxyl sulfate led to decreased ACh-induced relaxation in the aortic rings of female wild-type mice with normal renal function. Chu et al. [30] found that indoxyl sulfate impaired vasomotor responses, including increased PE-induced contraction and decreased ACh-induced relaxation in the aorta in five of six nephrectomized rats. In contrast, reduction of indoxyl sulfate by oral adsorbent AST-120 could normalize flow-mediated endothelium-dependent vasodilatation in patients with CKD [31], microvascular endothelial dysfunction (ACh-induced iontophoresis) in patients with CKD [32], and ACh-induced aortic relaxation in CKD mice [29]. However, few studies have investigated the direct association between indoxyl sulfate and vascular function, including vasorelaxation and vasocontraction induced by various stimuli, such as ligands and activators.

The present study aimed to investigate the effects of acute exposure of indoxyl sulfate on vascular function induced by various substances, such as ACh, nitric oxide (NO) donor sodium nitroprusside (SNP), noradrenaline, adenylyl cyclase activator forskolin, calcium ionophore (A23187), and transient receptor potential vanilloid 4 (TRPV4) agonist (GSK1016790A) in rat aorta using pharmacological approaches.

## 2. Results

### 2.1. Effects of Indoxyl Sulfate on Vasorelaxations Induced by ACh and SNP

**Figure 1:**
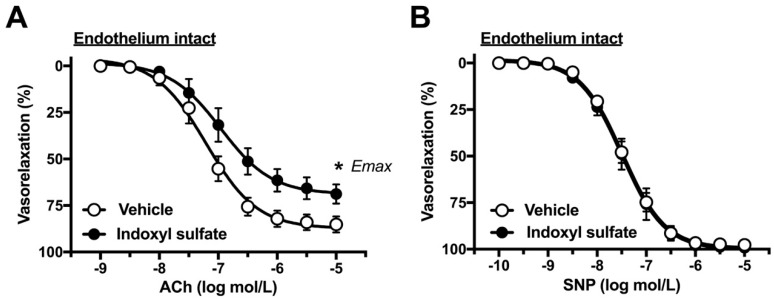
Concentration–response curves for acetylcholine (ACh) (**A**) or sodium nitroprusside (SNP) (**B**)-induced vasorelaxations in the aortas in the absence (vehicle) and presence of indoxyl sulfate (10^−4^ mol/L). Ordinate shows vasorelaxation as a percentage of PE-induced vasocontraction (0% being defined as the plateau level of precontraction). Data are presented as mean ± standard error of mean (SEM) values from ten (**A**) or five (**B**) experiments. * *p* < 0.05, vs. vehicle; E_max_.

In order to determine the direct acute effects of indoxyl sulfate on aortic function, the aortas were incubated with indoxyl sulfate (10^−4^ mol/L) for 30 min. First, we performed concentration–response curves for common endothelium-dependent or -independent vasodilators such as ACh (Figure 1A) or SNP, respectively (Figure 1B). As shown in Figure 1A, ACh-induced vasorelaxation was reduced in the aortas treated with indoxyl sulfate as compared with that in those treated with vehicle ((the maximal effect generated by the agonist (E_max_) %) 85.2 ± 4.2 (*n* = 10) vehicle vs. 68.9 ± 5.1 (*n* = 10) indoxyl sulfate (*p* < 0.05) and (a negative logarithm of EC_50_, which is the molar concentration of agonist producing 50% of the E_max _(pD_2_)) 7.22 ± 0.12 (*n* = 10) vehicle vs. 6.90 ± 0.14 (*n* = 10) indoxyl sulfate (*p* > 0.05)). In contrast, as shown in Figure 1B, SNP-induced vasorelaxation was similar between the two groups ((E_max_ %) 97.7 ± 1.4 (*n* = 5) vehicle vs. 99.1 ± 0.6 (*n* = 5) indoxyl sulfate (*p* > 0.05) and (pD_2_) 7.47 ± 0.08 (*n* = 5) vehicle vs. 7.47 ± 0.14 (*n* = 5) indoxyl sulfate (*p* > 0.05)).

### 2.2. Effects of Indoxyl Sulfate on Vasocontraction Induced by Noradrenaline and Isotonic High-K^+^

**Figure 2:**
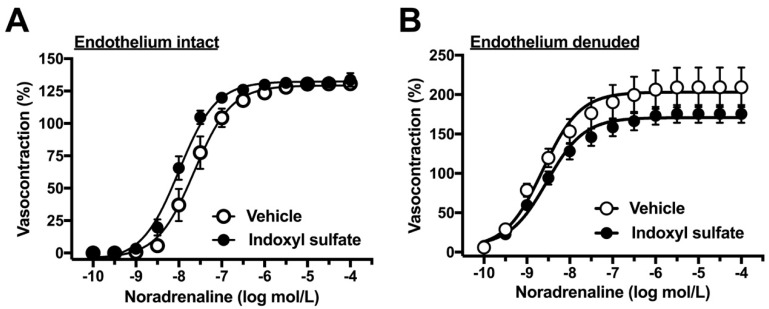
Concentration–response curves for noradrenaline-induced vasocontractions in endothelium-intact (**A**) or -denuded (**B**) aortas in the absence (vehicle) and presence of indoxyl sulfate (10^−4^ mol/L). The ordinate shows vasocontraction as a percentage of 80 mmol/L high-K^+^-induced vasocontraction. Data are presented as means ± standard error of mean (SEM) values from six experiments.

**Figure 3:**
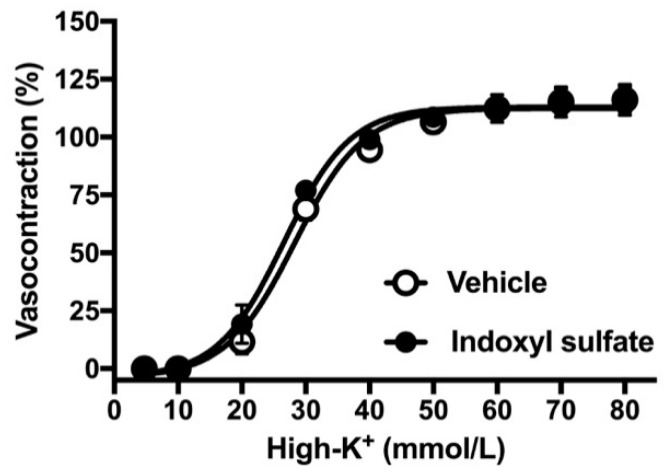
Concentration–response curves for high K^+^-induced vasocontractions in the aortas in the absence (vehicle) and presence of indoxyl sulfate (10^−4^ mol/L). The ordinate shows vasocontraction as a percentage of 80 mmol/L high-K^+^-induced vasocontraction. Data are presented as mean ± standard error of mean (SEM) values from eight experiments.

As the next step, we performed concentration–response curves for noradrenaline in the aorta of endothelium-intact or -denuded preparation. As shown in Figure 2A, the indoxyl sulfate-treated aorta had slightly but not significantly increased sensitivity to noradrenaline than the vehicle group in the endothelium intact preparation ((E_max_ %) 130.6 ± 3.9 (*n* = 6) vehicle vs. 133.8 ± 5.3 (*n* = 6) indoxyl sulfate (*p* > 0.05) and (pD_2_) 7.64 ± 0.15 (*n* = 6) vehicle vs. 8.01 ± 0.10 (*n* = 6) indoxyl sulfate (*p* = 0.07)). However, the noradrenaline-induced vasocontraction was not significant different between the vehicle and indoxyl sulfate groups in the endothelium-denuded preparation ((E_max _%) 209.4 ± 25.1 (*n* = 6) vehicle vs. 175.5 ± 11.2 (*n* = 6) indoxyl sulfate (*p* > 0.05) and (pD_2_) 8.64 ± 0.09 (*n* = 6) vehicle vs. 8.52 ± 0.12 (*n* = 6) indoxyl sulfate (*p* > 0.05)). As shown in Figure 3, high-K^+^-induced vasocontractions were unaffected by indoxyl sulfate ((E_max _%) 116.1 ± 6.7 (*n* = 8) vehicle vs. 116.3 ± 5.5 (*n* = 8) indoxyl sulfate (*p* > 0.05) and (EC_50_ mmol/L) 28.0 ± 1.6 (*n* = 8) vehicle vs. 26.0 ± 1.7 (*n* = 8) indoxyl sulfate (*p* > 0.05)).

### 2.3. Effects of Indoxyl Sulfate on Adenylyl Cyclase Activator-Induced Vasorelaxation

**Figure 4:**
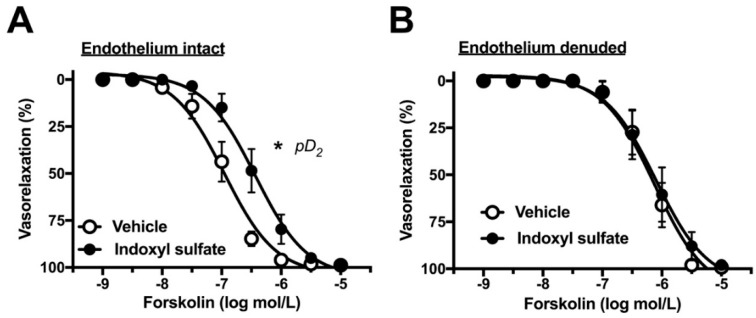
Concentration–response curves for forskolin-induced vasorelaxations in endothelium-intact (**A**) or -denuded (**B**) aortas in the absence (vehicle) and presence of indoxyl sulfate (10^−4^ mol/L). The ordinate shows vasorelaxation as a percentage of PE-induced vasocontraction (0% being defined as the plateau level of precontraction). Data are presented as mean ± standard error of mean (SEM) values seven (**A**) or eight (**B**) experiments. * *p* < 0.05, vs. vehicle; a negative logarithm of EC_50_, which is the molar concentration of agonist producing 50% of the E_max _(pD_2_).

In the next phase of the study, we investigated whether indoxyl sulfate affects cAMP-mediated vasorelaxation. For this, we performed concentration–response curves for forskolin, an adenylyl cyclase activator. As shown in Figure 4A, indoxyl sulfate-treated aortas reduced sensitivity to forskolin as compared with those from vehicle-treated aortas ((E_max_ %) 98.8 ± 1.0 (*n* = 7) vehicle vs. 98.4 ± 1.0 (*n* = 7) indoxyl sulfate (*p* > 0.05) and (pD_2_) 6.99 ± 0.11 (*n* = 7) vehicle vs. 6.43 ± 0.16 (*n* = 7) indoxyl sulfate (*p* < 0.05)). In endothelium-denuded preparations, forskolin-induced vasorelaxation was similar between the two groups ((E_max_ %) 99.3 ± 0.7 (*n* = 8) vehicle vs. 98.0 ± 1.9 (*n* = 8) indoxyl sulfate (*p* > 0.05) and (pD_2_) 6.12 ± 0.17 (*n* = 8) vehicle vs. 5.98 ± 0.23 (*n* = 8) indoxyl sulfate (*p* > 0.05)).

### 2.4. Effect of Indoxyl Sulfate on Calcium Ionophore- or TRPV4 Agonist-Induced Vasorelaxation

**Figure 5:**
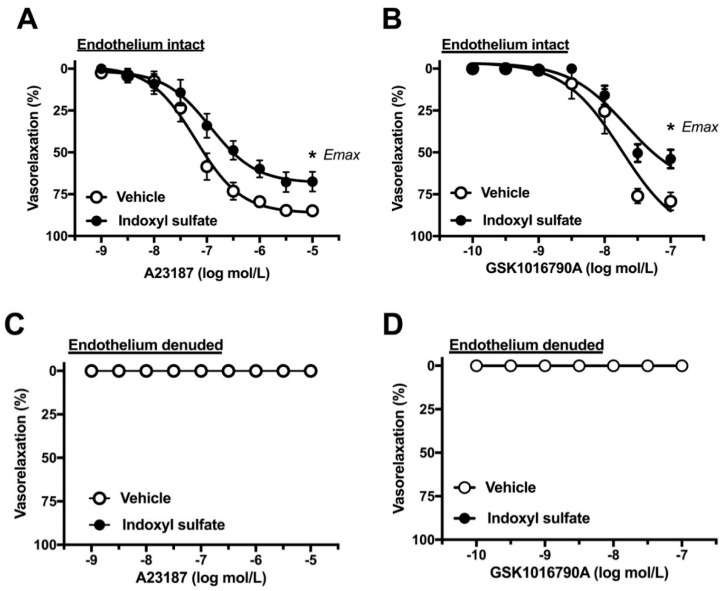
Concentration–response curves for A23187 (**A**) or GSK1016790A (**B**)-induced vasorelaxations in endothelium-intact (**A**,**C**) or -denuded (**B**,**D**) aortas in the absence (vehicle) and presence of indoxyl sulfate (10^−4^ mol/L). The ordinate shows vasorelaxation as a percentage of PE-induced vasocontraction (0% being defined as the plateau level of precontraction). Data are presented as mean ± standard error of mean (SEM) values from eight (**A**) or six (**B**–**D**) experiments. * *p* < 0.05, vs. Vehicle; E_max_.

Increased intracellular calcium level in the endothelium is a crucial event of endothelium-dependent relaxation [33,34]. We assessed whether indoxyl sulfate affects vasorelaxation induced by calcium modulators. As shown in Figure 5A, calcium ionophore A23187-induced vasorelaxation was significantly reduced in the indoxyl sulfate-treated group than in the vehicle-treated group ((E_max_ %) 86.5 ± 2.1 (*n* = 8) vehicle vs. 67.7 ± 5.8 (*n* = 8) indoxyl sulfate (*p* < 0.05) and (pD_2_) 7.24 ± 0.15 (*n* = 8) vehicle vs. 7.05 ± 0.25 (*n* = 8) indoxyl sulfate (*p* > 0.05)). As shown in Figure 5B, a TRPV4 agonist GSK1016790A-induced vasorelaxation was reduced more in the indoxyl sulfate-treated group than that in the vehicle-treated group ((E_max_ %) 79.2 ± 5.3 (*n* = 6) vehicle vs. 54.0 ± 5.5 (*n* = 6) indoxyl sulfate (*p* < 0.05) and (pD_2_) 7.79 ± 0.18 (*n* = 6) vehicle vs. 7.67 ± 0.07 (*n* = 6) indoxyl sulfate (*p* > 0.05)). Vasorelaxations induced by A23187 (Figure 5C) and GSK1016790A (Figure 5D) were eliminated by endothelium denudation in both vehicle and indoxyl sulfate groups.

### 2.5. Effect of Cell-Permeant SOD on ACh- or A23187-Induced Vasorelaxation

**Figure 6:**
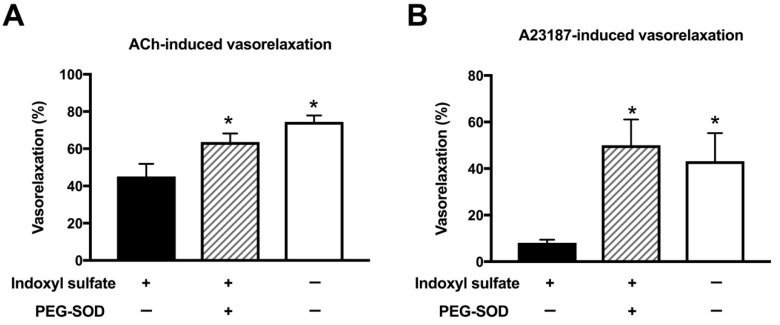
Effect of cell-permeant SOD on vasorelaxations induced by 3 × 10^−7^ mol/L acetylcholine (ACh) (**A**) or 10^−7^ mol/L A23187 (**B**) in the aorta treated with or without indoxyl sulfate. Polyethylene glycol-conjugated superoxide dismutase (PEG-SOD) (41 U/mL) was applied for 15 min; thereafter, indoxyl sulfate (10^−4^ mol/L) was incubated for 30 min before phenylephrine (PE) application. The ordinate shows vasorelaxation as a percentage of PE-induced vasocontraction (0% being defined as the plateau level of precontraction). Data are presented as mean ± standard error of mean (SEM) values from six experiments. * *p* < 0.05 vs. indoxyl sulfate group.

Superoxide is detrimental for vascular function [33,35]. We examined whether superoxide scavenger can normalize impaired endothelium-dependent relaxation in indoxyl sulfate-treated aortas. Indoxyl sulfate led to decreased vasorelaxations induced by a single application of ACh (3 × 10^−7^ mol/L) (Figure 6A) or A23187 (10^−7^ mol/L) (Figure 6B). A cell-permeant superoxide dismutase (SOD), polyethylene glycol-conjugated SOD (PEG-SOD) (41 U/mL) increased ACh- or A23187-induced vasorelaxations in indoxyl sulfate-treated aortas, suggesting that superoxide contributes to the impairment in endothelium-dependent relaxation.

### 2.6. Effect of Organic Anion Transporter Inhibitor on ACh- or A23187-Induced Vasorelaxation

**Figure 7:**
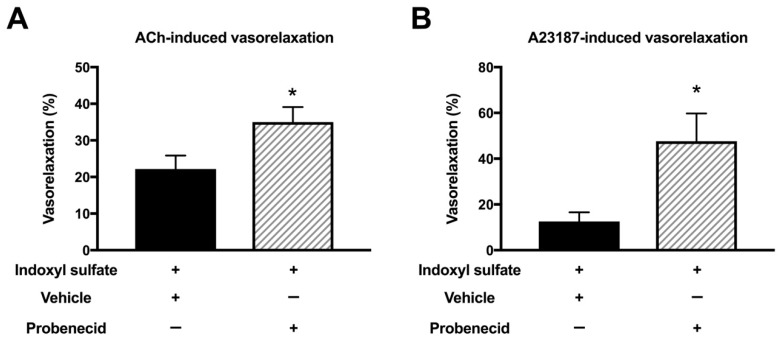
Effect of organic acid transporter inhibitor on vasorelaxations induced by 3 × 10^−7^ mol/L acetylcholine (ACh) (**A**) or 10^−7^ mol/L A23187 (**B**) in the aorta treated with indoxyl sulfate. Probenecid (10^−3^ mol/L) or vehicle (ethanol) was applied for 30 min; thereafter, indoxyl sulfate (10^−4^ mol/L) was incubated for 30 min before phenylephrine (PE) application. The ordinate shows vasorelaxation as a percentage of the PE-induced vasocontraction (0% being defined as the plateau level of precontraction). Data are presented as mean ± standard error of mean (SEM) values from six experiments. * *p* < 0.05 vs. indoxyl sulfate group.

It has been reported that indoxyl sulfate affects cellular function by transporting into the cells via an organic anion transporter [13,36,37]. Therefore, we studied the effect of an organic anion transporter inhibitor on the endothelium-dependent vasorelaxations in indoxyl sulfate-treated aortas. As shown in Figure 7, probenecid (10^−3^ mol/L) enhanced the vasorelaxations induced by ACh (3 × 10^−7^ mol/L) (Figure 7A) or A23187 (10^−7^ mol/L) (Figure 7B) in aortas treated with indoxyl sulfate.

### 2.7. Effect of NADPH Oxidase Inhibitor on Vasorelaxation in Indoxyl Sulfate-Treated Aorta

**Figure 8:**
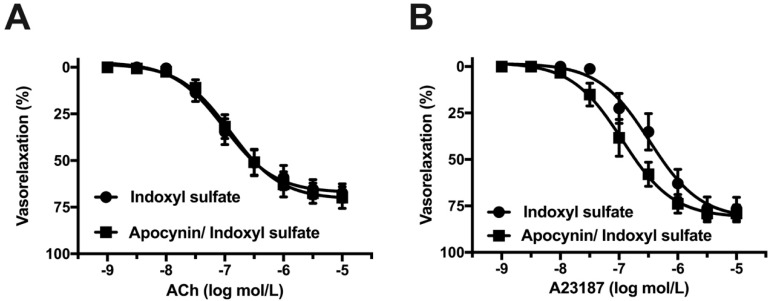

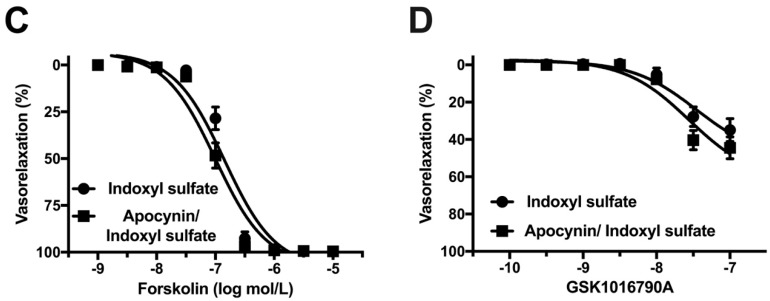
Effect of an NADPH oxidase inhibitor on vasorelaxations induced by acetylcholine (ACh) (**A**), A23187 (**B**), forskolin (**C**), or GSK1016790A (**D**) in the aorta treated with indoxyl sulfate. Apocynin (10^−4^ mol/L) or vehicle (DMSO) was applied for 30 min; thereafter, indoxyl sulfate (10^−4^ mol/L) was incubated for 30 min before phenylephrine (PE) application. The ordinate shows vasorelaxation as a percentage of the PE-induced vasocontraction (0% being defined as the plateau level of precontraction). Data are presented as mean ± standard error of mean (SEM) values from eleven (**A**), ten (**B**), and twelve (**C**,**D**) experiments.

NADPH oxidase plays a key source in vascular oxidative stress [38,39,40]. Therefore, we studied the effect of an NADPH oxidase inhibitor on the vasorelaxations in indoxyl sulfate-treated aortas. Figure 8 shows that apocynin (10^−4^ mol/L) did not affect the vasorelaxations induced by ACh (Figure 8A), A23187 (Figure 8B), forskolin (Figure 8C), or GSK1016790A (Figure 8D) in the aortas treated with indoxyl sulfate.

## 3. Discussion

Although indoxyl sulfate may have an effect on various phenomena in the vascular system, the direct relationship between acute indoxyl sulfate and vascular function, including vasorelaxation and vasocontraction, remains unclear. The findings of the present study showed that in vitro acute treatment with indoxyl sulfate for 30 min led to reduced ACh-induced relaxation, calcium ionophore-induced relaxation, cAMP-mediated relaxation, and TRPV4 agonist-induced relaxation in the rat aorta. Moreover, we found that the impairment of ACh- or calcium ionophore-induced relaxation by indoxyl sulfate could be prevented by superoxide scavenger or by an organic anion transporter inhibitor.

Few studies have investigated the direct acute association between indoxyl sulfate and vascular function. A seminal report by Six et al. [29] found that acute indoxyl sulfate led to concentration–dependent (i.e., 10^−4^ to 10^−3^ mol/L) impairments of ACh-induced relaxation in the aortic rings of female wild-type mice with normal renal function. In our study, we used indoxyl sulfate at 10^−4^ mol/L because several reports demonstrated that the concentration of indoxyl sulfate could induce deterioration of functions [16,41,42]. In our study, we observed vasorelaxation and vasocontraction induced by various substances that utilize various signaling cascades. When indoxyl sulfate was acutely incubated to the rat aortas, we observed the following responses. Vasorelaxations induced by ACh, an endothelium-dependent vasodilator [43], but not by SNP, a NO donor and endothelium-independent vasodilator [44], were impaired by indoxyl sulfate. These results suggested that acute exposure of indoxyl sulfate specifically impaired endothelium-mediated relaxation but did not influence cGMP-mediated vasorelaxation signaling in the aortic smooth muscle. These findings, which are acute exposure of indoxyl sulfate impairs ACh-induced endothelium-dependent vasorelaxation, are consistent with a report by Six et al. [29]. However, the lower concentration of indoxyl sulfate (i.e., 10^−4^ mol/L) were effective in the present study when compared to other reports [29], which may be explained by the difference between sex and species.

With regard to vasocontractile responses, no significant difference was found in the noradrenaline-induced vasocontraction in the presence or absence of indoxyl sulfate in both endothelium-intact and -denuded preparations of the rat aorta. Moreover, high-K+-induced vasocontraction was also unaffected by indoxyl sulfate. These results indicate that acute indoxyl sulfate did not have a significant effect on the contractile response in the rat aorta.

The relationship between indoxyl sulfate and cAMP-mediated vasorelaxation has not been fully elucidated. Reportedly, an activator of adenylyl cyclase forskolin led to vasorelaxation in both endothelium-dependent and -independent manners [45,46]. In our study, it is noteworthy that indoxyl sulfate reduced the sensitivity to forskolin-induced vasorelaxation in the endothelium-intact preparation but not endothelium-denuded preparation. These results suggest that a harmful effect of indoxyl sulfate on forskolin-induced vasorelaxation was resulting from alteration of endothelial function. Moreover, because no difference was found in forskolin-induced vasorelaxation with and without exposure of indoxyl sulfate in endothelium-denuded aortas, our data suggested that cAMP-mediated signaling in the aortic smooth muscle was not influenced by indoxyl sulfate. Several reports suggested that forskolin could activate eNOS in endothelial cells [45,47,48]. Thus, we suggest that the functional changes in endothelium-dependent vasorelaxation in the rat aorta acutely exposed with indoxyl sulfate may be due to a decrease in the production and/or release of NO as discussed below. 

The increment in intracellular calcium is an important event for endothelium stimulation and evokes endothelium-dependent relaxation. Therefore, in this study, we investigated the effects of indoxyl sulfate on vasorelaxation followed by increased level of intracellular calcium, such as a calcium ionophore (A23187) [49] and an agonist of TRPV4 (GSK1016790A) [50]. Indoxyl sulfate decreased vasorelaxations induced by both A23187 and GSK1016790A, suggesting that indoxyl sulfate may affect endothelial calcium regulation. Considering our data and the related evidence, acute treatment with indoxyl sulfate is detrimental for endothelial function rather than for vascular smooth muscle function.

Among the endothelium-derived relaxing factors (EDRFs), including NO, vasodilator prostaglandins and endothelium-derived hyperpolarizing factors, NO plays a pivotal role in regulating the vascular tone, especially, the large artery, such as the aorta [33,34]. The production of NO concomitant with eNOS activation is regulated by various factors, including precursor L-arginine, co-factors (e.g., tetrahydrobiopterin (BH4) and calmodulin), and post-translational modifications (e.g., phosphorylation and S-nitrosylation) [34]. In our present study, acute exposure of indoxyl sulfate impairs vasorelaxations not only by ACh but also by endothelial calcium elevations by A23187 and GSK106790A, and by endothelial cAMP elevation by forskolin. Thus, our results suggested that indoxyl sulfate may impair eNOS rather than specific upstream event of eNOS activation, including Ca2+/calmodulin pathway and cAMP/protein kinase A/eNOS phosphorylation pathway in the rat aorta. Stinghen et al. [51] found that indoxyl sulfate reduced NO production and increased ROS production, and the addition of BH4 had no effect on these parameters in a murine cerebral endothelial cell line. Tumur and Niwa [52] found that treatment with AST-120 to rats with chronic renal failure could restore the expression of glomerular eNOS and tubulointerstitial nNOS and NO production. However, how indoxyl sulfate modulates eNOS status, including coupling/uncoupling, post-translational modifications, and its expression remain unclear, necessitating further investigation on the point.

Moreover, endothelium-dependent NO-mediated relaxation defines not only NO production by eNOS, but also NO bioavailability, including destruction by ROS [33,34]. Redox signaling is also important for the regulation of NO bioavailability. In fact, Tumur and Niwa [53] observed that indoxyl sulfate inhibits NO production and cell viability by inducing oxidative stress in vascular endothelial cells. Moreover, using human umbilical vein endothelial cells, 1) Yu et al. [31] found that indoxyl sulfate induced ROS production, and pretreatment with antioxidants normalized the indoxyl sulfate-induced inhibition of proliferation, NO production, and inhibited senescence, and 2) Chou et al. [19] found that indoxyl sulfate increased the abundance of NADPH oxidase 4 (NOX4) and nuclear factor-kappa B. Furthermore, Chu et al. [30] found that impaired ACh-induced relaxation in the aorta of nephrectomized rats treated with indoxyl sulfate could be prevented by in vivo treatment with apocynin, tempol (SOD mimetic), mito-TEMPO (mitochondrial ROS scavenger), or fasudil (Rho kinase inhibitor). In our present study, impaired ACh- or A23187-induced vasorelaxations by indoxyl sulfate was increased by pretreatment with a cell-permeant superoxide scavenger, suggesting that impaired endothelium-dependent relaxation by indoxyl sulfate may be due to the reduction of NO bioavailability via increased superoxide. However, an inhibitor of NADPH oxidase did not increase vasorelaxations induced by ACh, A23187, forskolin, and GSK1016790A. Although our data suggested that activation of NADPH oxidase might not be a determinant for impaired vasorelaxations by indoxyl sulfate, further research is required to clarify molecular mechanisms as to how redox states in endothelial and smooth muscle cells and eNOS status in endothelial cells induced by acute indoxyl sulfate treatment may modulate in rat aortas.

Several reports have suggested that the circulatory levels of indoxyl sulfate increase in various pathophysiological states, such as CKD and diabetic nephropathy [54,55,56,57]. The concentrations of indoxyl sulfate in serum is approximately 2.5 × 10^−4^ to 3.6 × 10^−4^ mol/L [58,59] in patients with CKD, and its level elevates progressively with increased CKD stages [60]. In patients with advanced CKD, the level of total indoxyl sulfate exceeds 5 × 10^−4^ mol/L compared with approximately 10^−7^ to 2.4 × 10^−6^ mol/L in the healthy population [61]. The free form of indoxyl sulfate is approximately 10% of the total indoxyl sulfate in patients with CKD, whereas it is non-detectable in normal subjects [9]. In the present study, we used indoxyl sulfate at 10^−4^ mol/L for 30 min exposed to normal rat aortas, and our results suggest that indoxyl sulfate could impair endothelial function of normal animals even if it is short-term exposure. Indoxyl sulfate is the product of diet-derived tryptophan being converted by intestinal flora to indole and finally indoxyl sulfate in the body [58]. Therefore, the prevention of increased levels of indoxyl sulfate, for example, inhibition of production of indoxyl sulfate or its precursors in the gut may represent a potential approach to prevent endothelial dysfunction.

There were some limitations in the present study. Indoxyl sulfate can reportedly affect various functions after transport into the cells via an organic anion transporter [13,36,37]. In fact, our findings showed that impaired ACh- and A23187-induced vasorelaxations induced by indoxyl sulfate were increased by pretreatment with an organic anion transporter inhibitor. These data suggested that indoxyl sulfate led to endothelial dysfunction by transport into cells via an organic anion transporter. In addition, we could not rule out the possibility that indoxyl sulfate led to decrease of endothelium-dependent vasorelaxations, resulting from affected vascular smooth muscle cells because vascular smooth muscle was also a source of ROS. However, we could not determine the sites of action of indoxyl sulfate, such as the endothelial cells, vascular smooth muscle cells, or both, and the underlying molecular mechanisms. Further research needs to be conducted to achieve a deeper understanding of this subject as well. 

In conclusion, our data found that indoxyl sulfate directly impairs endothelium-dependent vasorelaxation induced by various substances in the rat aorta. The preventions of indoxyl sulfate elevation in the body and of cell transportation are important for the maintenance of vascular health.

## 4. Materials and Methods

### 4.1. Animals

Male Wistar rats were purchased from the Japan Laboratory Animals. Inc., Tokyo, Japan and housed in a pathogen-free facility. This study was approved by the Hoshi University Animal Care and Use Committee that has been accredited by the Ministry of Education, Culture, Sports, Science, and Technology of Japan (permission code: 29-135, permission date: 21 June 2018).

### 4.2. Vascular Function Study

Rats were sacrificed via exsanguination from the abdominal aorta under isoflurane anesthesia, and the thoracic aorta was carefully and rapidly isolated and placed in an ice-chilled, oxygenated, modified Krebs–Henseleit solution (KHS; consisting (in mM) of 118.0 NaCl, 4.7 KCl, 25.0 NaHCO_3_, 1.8 CaCl_2_, 1.2 NaH_2_PO_4_, 1.2 MgSO_4_, and 11.0 glucose). Each aorta was separated from the surrounding connective tissue and fat, cut into rings, and then mounted on the organ bath system. Subsequently, the vascular function was assessed by measuring the vascular isometric force, as reported previously [62,63,64]. To investigate the effect of indoxyl sulfate on vasorelaxation, each substance (ACh [10^−9^–10^−5^ mol/L], SNP [10^−10^–10^−5^ mol/L], forskolin [10^−9^–10^−5^ mol/L], A23187 [10^−9^–10^−5^ mol/L], or GSK1016790A [10^−10^–10^−7^ mol/L]) was cumulatively applied after plateau contraction was achieved with 10^−6^ mol/L phenylephrine (PE) of the aorta incubated with indoxyl sulfate (10^−4^ mol/L for 30 min) or vehicle (ultrapure water).

To investigate the effect of indoxyl sulfate on vasocontraction, the aortic rings were incubated with 10^−4^ mol/L indoxyl sulfate for 30 min; thereafter, noradrenaline (10^−10^–10^−4^ mol/L) or high-K^+^ (10–80 mmol/L) was cumulatively applied. In some experiments, the endothelium-denuded aortas were achieved by gently rubbing the lumen side of the vessels using a pipette tip, as well as previous papers [62,63,64]. Endothelium integrity was assessed by contracting the aortic segments with 10^−6^ mol/L PE, followed by stimulation with ACh (10^−6^ mol/L for endothelium intact ring or 10^−5^ mol/L for endothelium-denuded ring).

In order to investigate the effect of SOD on vasorelaxations in the indoxyl sulfate-treated aorta, the aorta was preincubated with PEG-SOD (41 U/mL) or vehicle (ultrapure water) for 15 min; thereafter indoxyl sulfate (10^−4^ mol/L) or vehicle was applied for 30 min before PE application. Following this, ACh (3 × 10^−7^ mol/L) or A23187 (10^−7^ mol/L) was applied.

To investigate the effect of probenecid on vasorelaxations in the indoxyl sulfate-treated aorta, the aorta was preincubated with probenecid (10^−3^ mol/L) or vehicle (ethanol) for 30 min following the application of indoxyl sulfate (10^−4^ mol/L) for 30 min before PE application. Thereafter, ACh (3 × 10^−7^ mol/L) or A23187 (10^−7^ mol/L) was applied.

To investigate the effect of apocynin on vasorelaxations in the indoxyl sulfate-treated aorta, the aorta was preincubated with apocynin (10^−4^ mol/L) or vehicle (DMSO) for 30 min following the application of indoxyl sulfate (10^−4^ mol/L) for 30 min before PE application. Thereafter, ACh (10^−9^–10^−5^ mol/L), A23187 (10^−9^–10^−5^ mol/L), forskolin (10^−9^–10^−5^ mol/L), or GSK1016790A (10^−10^–10^−7^ mol/L) was cumulatively applied.

In pretreatment with drugs before PE application, vehicle and drugs were applied in appropriate volumes of corresponding groups to an organ bath containing KHS with continuously gassed with 95% O_2_, 5% CO_2_ at 37 °C.

### 4.3. Statistical Analyses

The results are expressed as means ± standard error of mean values. Each vasorelaxation is expressed as a percentage of the PE-induced vasocontraction. Vasocontraction is expressed as a percentage of the response to 80 mmol/L high-K^+^. Statistical evaluations between the two groups were performed using Student’s *t*-test, and one-way analysis of variance (ANOVA) followed by Dunnett test was used for comparisons among the three groups. A value of *p* < 0.05 was considered statistically significant.
